# Antidepressant Effects of Repetitive Transcranial Magnetic Stimulation Over Prefrontal Cortex of Parkinson's Disease Patients With Depression: A Meta-Analysis

**DOI:** 10.3389/fpsyt.2018.00769

**Published:** 2019-01-29

**Authors:** Liang Zhou, Zhiwei Guo, Guoqiang Xing, Haitao Peng, Mengjie Cai, Huaping Chen, Morgan A. McClure, Lin He, Liangwen Xiong, Bin He, Fei Du, Qiwen Mu

**Affiliations:** ^1^Department of Radiology and Imaging Institute of Rehabilitation and Development of Brain Function, The Second Clinical Medical College of North Sichuan Medical College Nanchong Central Hospital, Nanchong, China; ^2^School of Clinical Medicine,The Clinical Medical College of Southwest Medical University, Luzhou, China; ^3^Lotus Biotech.com LLC., John Hopkins University-MCC, Rockville, MD, United States; ^4^Department of Genitourinary, University of Texas MD Anderson Cancer Center, Houston, TX, United States; ^5^Department of Psychiatry, Harvard Medical School, Belmont, CA, United States; ^6^Department of Radiology, Peking University Third Hospital, Beijing, China

**Keywords:** repetitive transcranial magnetic stimulation, prefrontal cortex, Parkinson's disease, depression, meta-analysis

## Abstract

**Objective:** The purpose of this meta-analysis was to investigate the antidepressant effects of repetitive transcranial magnetic stimulation (rTMS) over the prefrontal cortex (PFC) of patients with Parkinson's disease (PD) and to determine the optimal rTMS parameters, such as the intensity, frequency and the delivered pattern of rTMS stimulation.

**Methods:** EMBASE, PubMed, Web of Science, MEDLINE, and Cochrane data bases were researched for papers published before March 12, 2018. Studies investigating the anti-depression effects of rTMS over PFC in patients with PD were considered. The main outcomes of pre- and post-rTMS treatment as well as score changes were all extracted. The mean effect size was estimated by calculating the standardized mean difference (SMD) with 95% confidence interval (CI) by using fixed or random effect models as appropriate.

**Results:** Nine studies containing 137 PD patients with depression were included. The pooled results showed significant pre-post anti-depressive effects of rTMS over PFC in PD patients with depression (SMD = −0.80, *P* < 0.00001). The subgroup analyses of stimulation intensity, frequencies, and models also revealed significant effects (Intensities: 90% RMT: SMD = −1.16, *P* = 0.0006; >100% RMT: SMD = −0.82, *P* < 0.0001. Frequencies: < 1.0 Hz: SMD = −0.83, *P* = 0.03; 5.0 Hz: SMD = −1.10, *P* < 0.0001; ≥10.0 Hz: SMD = −0.55, *P* = 0.02. Models: Continuous: SMD = −0.79, *P* < 0.0001; Discontinuous: SMD = −0.84, *P* = 0.02). But the results of the studies with place-controlled designs were not significant (Overall: SMD = −0.27, *P* = 0.54. Intensities: 90% RMT: SMD = 0.27, *P* = 0.68; 100% RMT: SMD = −0.32, *P* = 0.33. Frequencies: 5.0 Hz: SMD = −0.87, *P* = 0.10; ≥10.0 Hz: SMD = 0.27, *P* = 0.66. Models: Continuous: SMD = −0.28, *P* = 0.68; Discontinuous: SMD = −0.32, *P* = 0.33). The greater effect sizes of rTMS with 90% RMT, 5.0 Hz in discontinuous days can be observed rather than the other parameters in both kinds of analyses across study design.

**Conclusions:** rTMS may have a significant positive pre-post anti-depressive effect over PFC on patients with depression, especially by using 5.0 Hz frequency with 90% RMT intensity in discontinuous days, which may produce better effects than other parameters. The real effect, though, was not different from that of the placebo. Future studies with larger sample sizes and high-quality studies are needed to further corroborate our results and to identify the optimal rTMS protocols.

## Introduction

Although Parkinson's disease (PD) is considered a movement disorder, a significant proportion of PD patients has also suffered from non-motor symptoms (1, 2). For example, about 35% of PD patients may have depressive symptoms, 17% may suffer major depressive disorder and 13% may suffer dysthymia (3). The severity and incidence of these symptoms rise with the development of PD—which negatively affects quality of life—the progression of overall disability (4), and are not related to the progression of motor symptoms of PD patients (5). These non-motor symptoms not only exert a vital effect on quality of life in patients, but also increase caregiver burden and health care costs. Although some newer dopamine agonists have an anti-depressive effect (6), most of the treatment with Levodopa does not relieve depressive symptoms (7).

Depending on the principle of electromagnetic induction, repetitive transcranial magnetic stimulation (rTMS) realizes a painless, non-invasive stimulation on the cerebral cortex that is well tolerated. (8). The basic mechanism in using rTMS over the prefrontal cortex (PFC) is that left dorsolateral prefrontal cortex (DLPFC) hypo-activity has been featured as an important role in the pathophysiological process of depression (9, 10), and the improvement of its activity has been related to symptom alleviation (11). High-frequency rTMS (>1.0 Hz, HF-rTMS) could increase cortical excitability, while low-frequency rTMS (≤1.0 Hz, LF-rTMS) could decrease it (12). Some studies have shown antidepressant effects of rTMS on major depression (MD) patients (13, 14). Cardoso et al. observed a large antidepressant effect from 5.0 Hz rTMS (15) that was superior to other reports that used 10.0 or 20.0 Hz of rTMS (16). In addition, a meta-analysis has proved that the same therapeutic effect for MD could be acquired in both HF-rTMS and LH-rTMS patterns (17). Hence, we hypothesized that rTMS may have a similar antidepressant effect on depressed PD patients.

Recent studies have shown that depressed PD patients who had an antidepressant reaction to rTMS treatment had improved activity in the cingulate gyrus when compared to the PD patients without showing antidepressant response to rTMS treatment (18, 19). Furthermore, studies have also shown that rTMS over left DLPFC (20, 21) or both left and right DLPFC (22) was effective in PD patients with depression and rTMS had antidepressant effects based on the Beck Depression Inventory (BDI) and Hamilton Rating Scale for Depression (HRSD) scores when compared with selective serotonin re-uptake inhibitors (SSRIs) (23). These divergent results in literature may arise from different designs of rTMS protocols such as the intensity, frequency, and the delivered pattern of rTMS stimulation (24). Although several meta-analyses on the topic of rTMS for depressed PD patients have been published, they showed only that rTMS was superior to sham-rTMS and that they had similar antidepressant efficacy as SSRIs, but had not clearly indicated which mode of rTMS represented the optimal parameters (23, 25). This meta-analysis was aimed to extend these findings and examine the efficacy of rTMS over PFC on depression in depressed PD patients, and we sought to determine the influence of the intensity of the RMT, its frequency, and the delivered pattern of rTMS stimulation.

## Methods

### Search Strategy

We systematically searched studies that were undertaken to identify the effects of rTMS over PFC on depression in PD patients in the EMBASE, PubMed, MEDLINE, and Cochrane databases published before March 12, 2018. The combinations of key terms included “Parkinson's disease” OR “Parkinson disease” OR “PD” AND “transcranial magnetic stimulation” OR “repetitive transcranial magnetic stimulation” OR “TMS” AND “depression” and their synonyms were used for each database.

### Study Selection

The inclusion criteria used were population (depressive patients diagnosed with PD), intervention (rTMS over PFC), comparison (sham-controlled group or condition), outcomes of depression, and language (published in English). Studies were excluded if there was no data available for the calculation of the effect size. There was no limit on the designs of trials. Randomized controlled trials (RCTs), cross-over trials, as well as self-contrast trials, were all included in the search.

### Quality Assessment of the Study

The methodological quality of each chosen study was assessed in accordance with the suggestions by Moher et al. (26) and Cochrane Collaboration (27) with slight modifications which included four aspects: (1) control design: “1” represents that the trial groups were compared with healthy groups, “2” represents patient controls, and “3” represents both controls; otherwise, it was marked as “0”; (2) randomization: if the subjects were randomly assigned to different groups it is marked as “1”, and if not it is marked as “0”; (3) blind process: marked as “0 - 2” (a non-blind or non-described process recorded as “0,” single-blind as “1,” and double-blind process as “2,”); and (4) dropout number: marked as the number of subjects who dropped out of the rTMS treatment.

### Data Extraction

Two independent reviewers carried out data extraction. Any disagreements were resolved by discussing with a third reviewer. Relevant data included the pivotal constituent of the general research information, the characteristics of study subjects, rTMS protocol, and depression scale scores of outcomes (including the scale scores of pre- and post-treatment as well as score changes of experimental and control group patients). The mean value and standard deviation (SD) of the outcome assessments were extracted for further analysis. If the standard error of the mean (SEM) was provided in the article, it was transformed to the standard deviation (SD) by using the formula of SD = SEM$\times \sqrt{n}$ (28). We contacted the authors of the research to obtain additional data if the study information was inadequate.

### Data Synthesis and Analysis

All statistical analyses of this meta-analysis were conducted by using Review Manager 5.3 (Cochrane Collaboration, Oxford, England) with a significance of *p* < 0.05. The standardized mean difference (SMD) was applied to the effect size with a 95% confidence interval (CI) as the significant results. The effect model was determined by the heterogeneity which was assessed by using the Cochran's Q statistic and *I*^2^ test (29). If heterogeneity was not significant (the Q test showed a *p* > 0.05 or *I*^2^ < 50%), the fixed-effect model was applied in this meta-analysis. Otherwise, the random-effect was used if the heterogeneity was found (the Q test displayed *p* < 0.05 or *I*^2^ ≥ 50%). Differences between groups were assessed by partitioning heterogeneity and using the χ^2^ distribution with degrees of freedom (*df*). Subgroup analyses were undertaken to probe into the source of the heterogeneity and investigate the effect differences between different rTMS treatment protocols including frequency, RMT, etc. The depression scale score changes could better reflect the effect of rTMS on patients than the score of post-rTMS treatment. Therefore, the change scores were preferentially selected and were used in this meta-analysis. Also, the analyses that only used the scale score of pre- and post-treatment as well as those with change scores alone were conducted to reduce heterogeneity and to find the pre-post differences as well as the effects different from placebos.

### Risk of Publication Bias

By considering that positive outcomes can be published more easily than negative outcomes, selective omission exists in the literature. Also, studies with insufficient data may have been excluded according to the inclusion criteria. Publication bias of this meta-analysis may exist due to both these situations. Hence, the risk of publication bias was assessed by using a funnel plot. It was deemed that there was no substantial publication bias in the meta-analysis if the inverse funnel plot was almost symmetrical and most of studies were distributed in its superior part (30). The risk of bias of the selected studies was assessed by two independent reviewers.

## Result

### Study Selection and Inclusion

**Figure 1:**
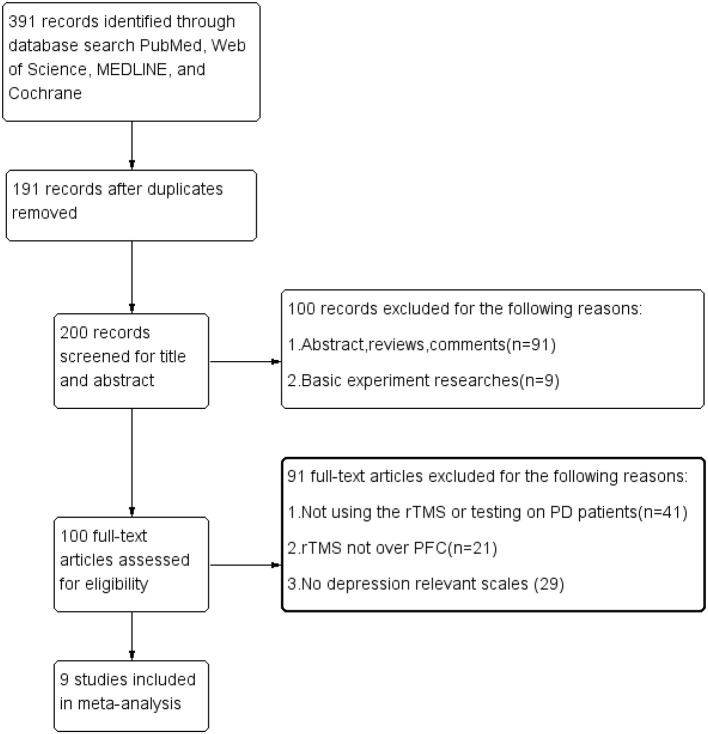
Flow diagram.

In total, 391 articles were found from the database search, and 191 were excluded on account of the replication. After reviewing the titles and abstracts, 100 studies were not included because they did not conform to the inclusion standards. By scanning the full content of the rest of the articles, 91 studies were further eliminated for not having evaluated the effects of rTMS over the PFC or for a lack of relevant result measurements of depression. Finally, only nine trials were included in our meta-analysis. The procedure of the selection is summarized in Figure 1.

### Characteristics and Quality of the Included Studies

**Figure 2:**
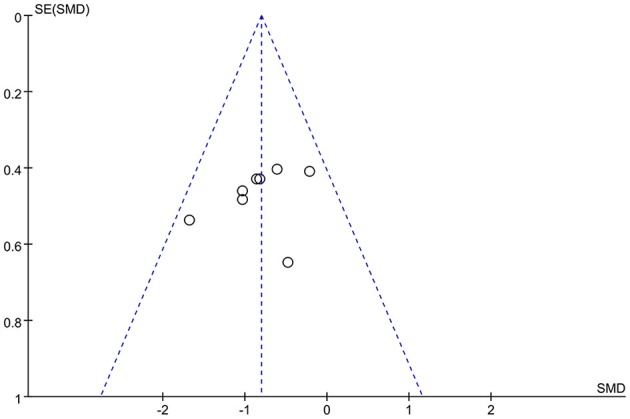
The risk of bias assessment of each study.

Two of the nine studies that met the inclusion criteria stated that they used open study designs (22, 31), five studies were analyzed and compared pre- and post-control (15, 22, 31–33), and four studies were analyzed and compared between experimental and control groups, three of which (all except for 33) also provided the pre- and post-treatment depression scale scores for each group (21, 34–36). Four studies evaluated the effects of greater than or equal to 10.0 Hz rTMS (31, 32, 35, 36), three studies used 5.0 Hz rTMS (15, 21, 34), and two studies used less than 1.0 Hz (22, 33). In terms of the stimulation intensity in each session of rTMS treatment, one study (36) did not describe the RMT in the article and we did not get any response after contacting the author to obtain the study information. Two studies used an intensity of 90% RMT for each session (21, 34), one study 100% of the RMT (36), and the rest of the five studies used the supra-threshold of the RMT (15, 22, 31–33). Furthermore, as for continuous or discontinuous days, the rTMS treatments that were repeated in consecutive days are ascribed to continuous days, except for the weekends and moments when the subjects experienced an interval of at least 1 day without stimulation ahead of the next session. These days were ascribed to discontinuous days. Six studies carried out rTMS treatment in continuous days (21, 22, 31, 32, 34, 35) and three studies carried out rTMS in discontinuous days (15, 33, 36). The study parameters and the characteristics of participants in the nine included studies are summarized in Tables 1, 2. The quality assessment of the study is summarized in Table 3. The risk of bias assessment of each study is summarized in Figure 2. The inverse funnel plot is almost symmetrical and most of the studies were distributed in its superior part. Therefore, it is deemed that there was no substantial publication bias in this meta-analysis.

**Table 1:** Basic characteristics of included studies.

**References**	**Design**	**Sample size**	**Age (Mean ± SD)**	**Sex (M/F)**	**Disease duration (Mean ± SD)**	**Session/duration**
Cardoso et al. 15	RCTs	11	67 ± 8.3	NR	11 ± 7.6	12 days/4 weeks
Boggio et al. (32)	RCTs	13	NR	NR	NR	10 days/2 weeks
Brys et al. (35)	RCTs	Ne:12 Nc:15	Ne:64.6 ± 12.3 Nc:64.0 ± 7.4	Ne:6/6 Nc:11/4	Ne: 7.7 ± 4.2 Nc: 4.5 ± 2.2	10 days/2 weeks
Epstein et al. (31)	Self-control	12	NR	9/5	NR	10 days/2 weeks
Pal et al. (34)	RCTs	Ne:12 Nc:10	Ne: 68.5 Nc: 67.5	Ne:6/6 Nc:5/5	NR	10 days /2 weeks
Shin et al. (21)	RCTs	Ne:10 Nc:8	Ne: 69 Nc: 67	Ne:6/4 Nc:2/6	NR	10 days/2 weeks
Furukawa et al. (33)	Self-control	5	66.83 ± 3.43	3/3	7.17 ± 3.06	3 months
Dragasevic, et al. (22)	Self-control	10	59.9 ± 8.5	6/4	NR	10 days/2 weeks
Yokoe et al. (36)	RCTs	19	69.1 ± 8.4	7/12	9.5 ± 3.2	3 days/4 weeks

*M, male; F, female; Ne, number of participants in experimental group; Nc, number of participants in control group; NR, no report*.

**Table 2:** Study characteristics of rTMS stimulation protocols.

**References**	**Stimulation parameter**					**Continuous/discontinuous days**	**Data (Mean ± SD)**	**Depression scale**
	**Frequency**	**Intensity**	**Coil type**	**Sessions-pulse**	**Site**		
Cardoso et al. (15)	5.0 Hz	120% RMT	F8	3,750 × 12	Left DLPFC	Discontinuous	pre: 23.36 ± 5.89 post: 14.91 ± 9.51	BDI
Boggio et al. (32)	15.0 Hz	110% RMT	F8	3,000 × 10	Left DLPFC	Continuous	pre: 24.5 ± 10.82 post: 17.9 ± 10.10	BDI
Brys et al. (35)	10.0 Hz	NR	F8	2,000 × 10	Left DLPFC	Continuous	real: −1.4 ± 6.5 sham: −6.1 ± 3.4	HAM-D
Epstein et al. (31)	10.0 Hz	110% RMT	F8	1,000 × 19	Left DLPFC	Continuous	pre: 28.6 ± 8.77 post: 21.3 ± 7.66	BDI
Pal et al. (34)	5.0 Hz	90% RMT	F8	600 × 10	Left DLPFC	Continuous	real: 5.17 ± 3.66 sham: 7.7 ± 6.7	BDI
Shin et al. (21)	5.0 Hz	90% RMT	F8	600 × 10	Left DLPFC	Continuous	real: −6.17 ± 3.55 sham: −0.97 ± 3.16	BDI
Furukawa et al. (33)	0.2 Hz	120% RMT	F8	100 × 12	Bilateral DLPFC	Discontinuous	pre: 63.2 ± 10.8 post: 56.1 ± 16.1	SDS
Dragasevic et al. (22)	0.5 Hz	110% RMT	F8	100 × 10	Bilateral PFC	Continuous	pre: 21.1 ± 4.7 post: 16.0 ± 4.8	BDI
Yokoe et al. (36)	10.0 Hz	100% RMT	F8	1,000 × 11	Bilateral DLPFC	Discontinuous	real: −1.53 ± 7.02 sham: 0.63 ± 6.33	SDS

*RMT, rest motor threshold; NR, no report; DLPFC, dorsolateral prefrontal cortex; PFC, prefrontal cortex; Pre, pre-depression recovery; Post, post-depression recovery; BDI, Beck depression inventory; HAM-D, Hamilton rating scale for depression; SDS, self-rating depression scale*.

**Table 3:** Quality assessment of the included studies.

**References**	**Randomization**	**Blind procedure**	**Control design**	**Descriptions of baseline demographic and clinical characteristics**	**Dropout number**	**Adverse effects**
Cardoso et al. (15)	1	1	2	1	0	U
Boggio et al. (32)	1	2	2	0	0	0
Brys et al. (35)	1	2	2	1	1	34
Epstein et al. (31)	0	0	0	1	2	0
Pal et al. (34)	1	2	2	1	0	2
Shin et al. (21)	1	1	2	1	0	0
Furukawa et al. (33)	0	0	0	1	0	0
Dragasevic et al. (22)	0	0	0	0	0	0
Yokoe et al. (36)	1	2	1	1	0	1

*U, unclear, insufficient information to categorize*.

### Meta-Analyses

#### Overall Effects

**Figure 3:**
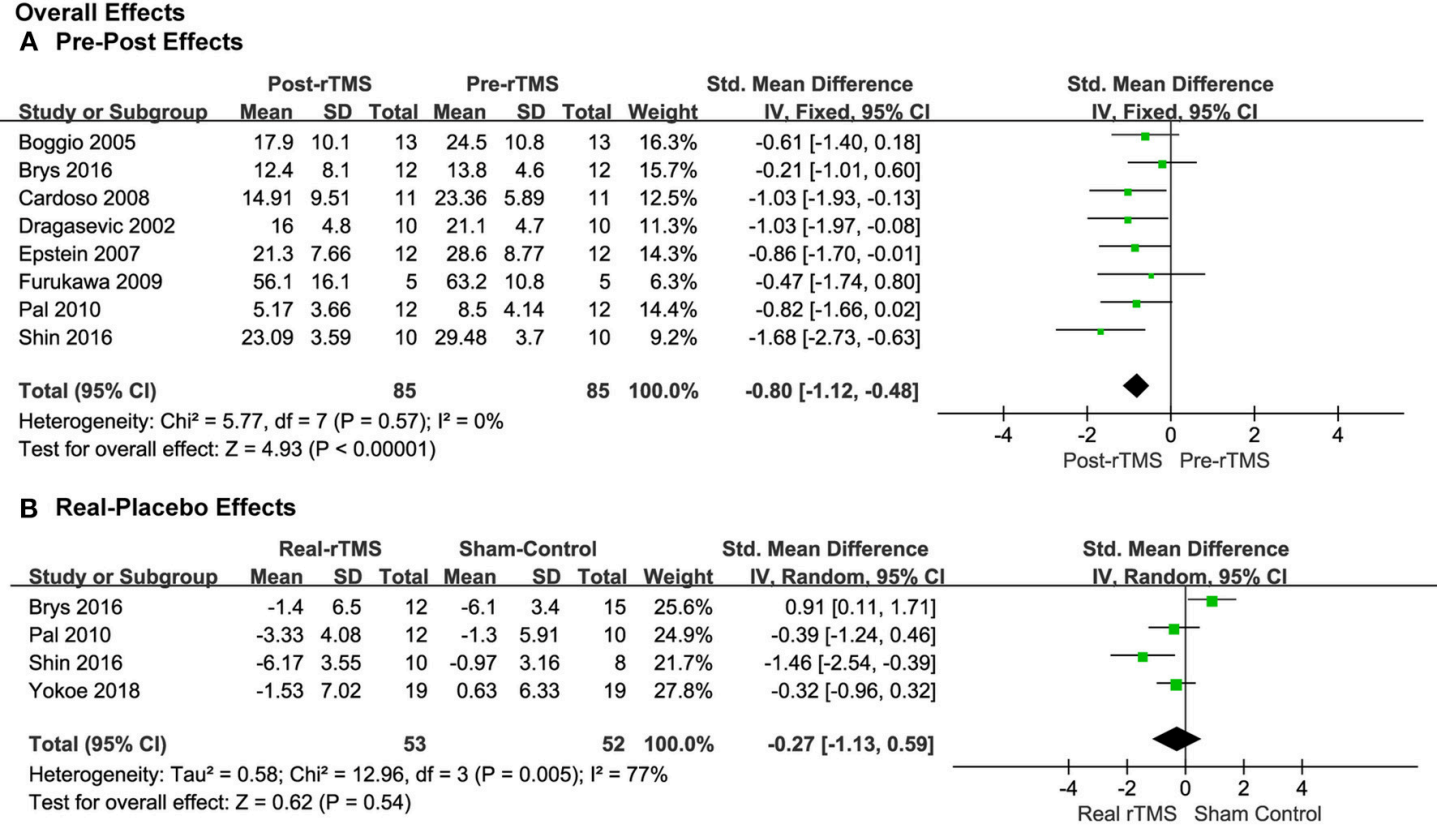
Forest plots of overall effects showed a significant pre-post anti-depressive effect **(A)** and non-significant real-placebo effect **(B)**.

Eight studies provided the pre- and post-treatment depression scale scores for the patients of the rTMS group. The pooled results from the analysis of the eight studies with pre-post treatment designs revealed a significant overall anti-depressive effect of rTMS therapy over PFC on the depression outcome scores (SMD = −0.80, 95% CI, −1.12 to −0.48, *P* < 0.00001; Figure 3A). The results of the four studies using placebos as controls, though, revealed that the effect size was not significant (SMD = −0.27, 95% CI, −1.13 to 0.59, *P* = 0.54; Figure 3B), with significant heterogeneity, which is different with the pre-post treatment effect.

#### Comparison of Stimulation Frequencies

**Figure 4:**
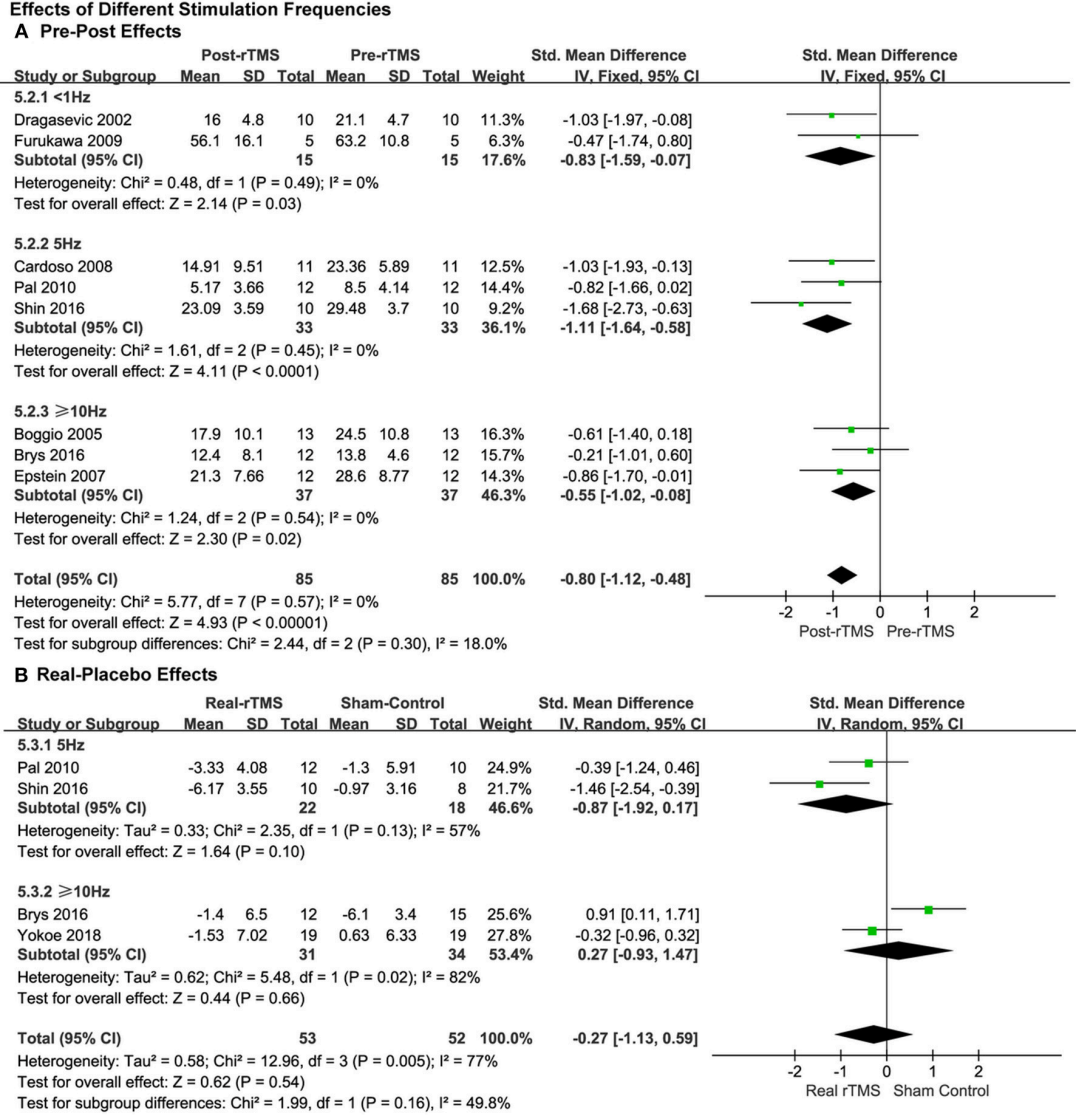
Comparison of the anti-depressive effects of different stimulation frequencies (<1.0 vs. 5.0 vs. ≥10.0 Hz) for the studies with pre-post treatment design **(A)** and place-controlled design **(B)**.

Studies that applied less than 1.0, 5.0, and ≥10.0 Hz of rTMS treatment also produced significant pre-post antidepressant effects (SMD = −0.83, 95% CI, −1.59 to −0.07, *P* = 0.03; SMD = −1.11, 95% CI, −1.64 to −0.58, *P* < 0.0001; SMD = −0.55, 95% CI, −1.02 to −0.08, *P* = 0.02; Figure 4A), respectively, without significant heterogeneity in the studies with pre-post treatment design.

This was the same as the subgroup analysis of stimulation intensity. No significant effects of rTMS were observed in the studies with place-control design, whether 5.0 Hz rTMS (SMD = −0.87, 95% CI, −1.92 to 0.17, *P* = 0.10; Heterogeneity test: Chi^2^ = 2.35, *P* = 0.13, *I*^2^ = 57%; Figure 4B) or ≥10.0 Hz of rTMS (SMD = 0.27, 95% CI, −0.93 to 1.47, *P* = 0.66; Heterogeneity test: Chi^2^ = 5.48, *P* = 0.02, *I*^2^ = 82%; Figure 4B) was used.

#### Effect of Different Stimulation Intensities

**Figure 5:**
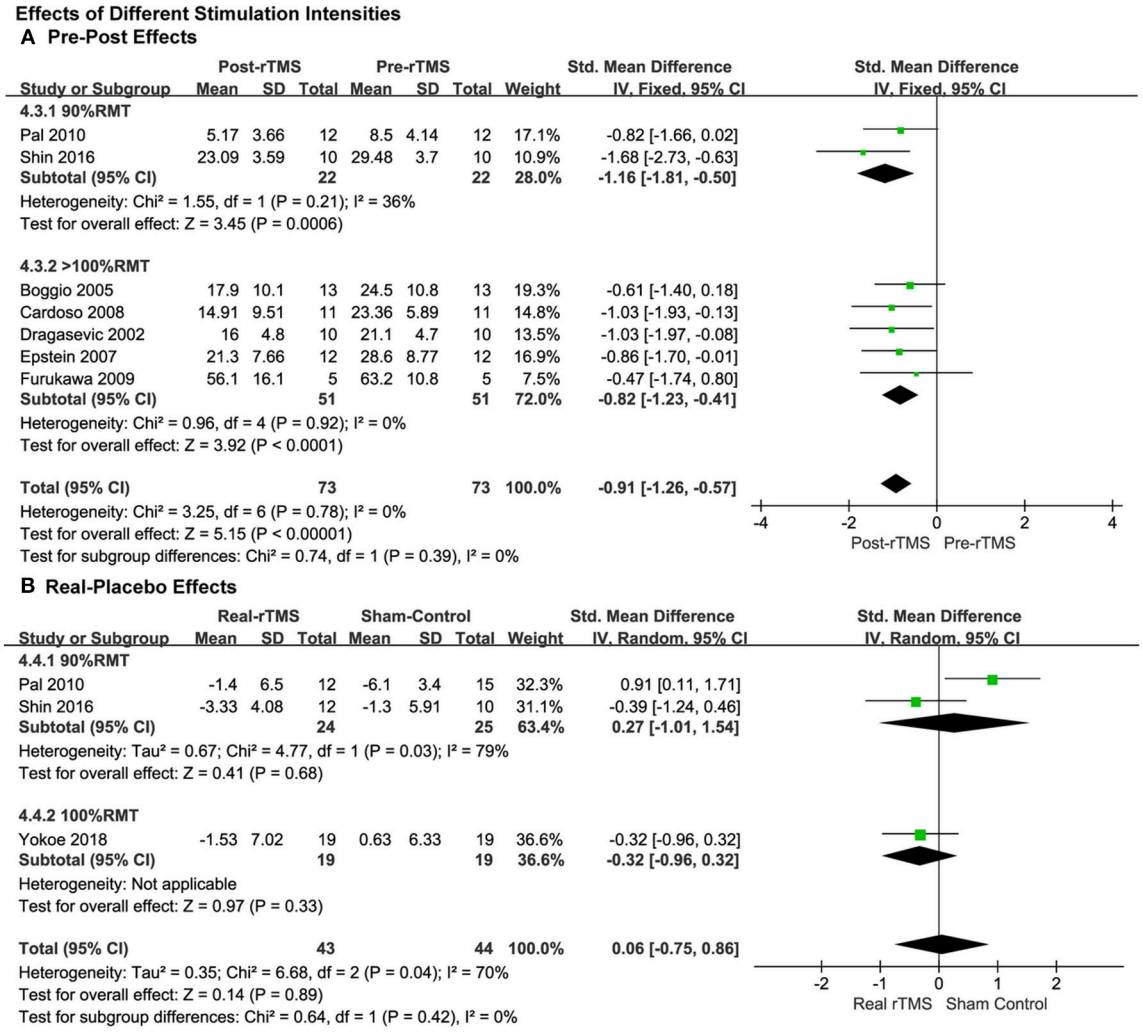
Comparison of the anti-depressive effects of different stimulation intensities (90% RMT vs. 100% RMT vs. ≥100% RMT) for the studies with pre-post treatment design **(A)** and place-controlled design **(B)**.

For the analysis of the studies with pre-post treatment design, the pooled results of the five studies by using a supra-threshold (>100% RMT) stimulation intensity showed a significant anti-depressive pre-post effect (SMD = −0.82, 95% CI, −1.23 to −0.41, *P* < 0.0001; Figure 5A), which was also observed in the studies with an intensity of 90% RMT (SMD = −1.16, 95% CI, −1.81 to −0.50, *P* = 0.0006; Figure 5A), and the effect size was greater than the supra-threshold group. No significant heterogeneity was in observed either of the two groups.

The subgroup analysis of stimulation intensity in the studies with a placebo control design was conducted as well. The stimulation intensities of 90% RMT and 100% RMT were used in the three included studies. The supra-threshold (>100% RMT) intensity was not used. No significant effects were observed in either group (90% RMT: SMD = 0.27, 95% CI, −1.01 to 1.54, *P* = 0.68; 100% RMT: SMD = −0.32, 95% CI, −0.96 to 0.32, *P* = 0.33; Figure 5B).

#### Continuous vs. Discontinuous Treatment

**Figure 6:**
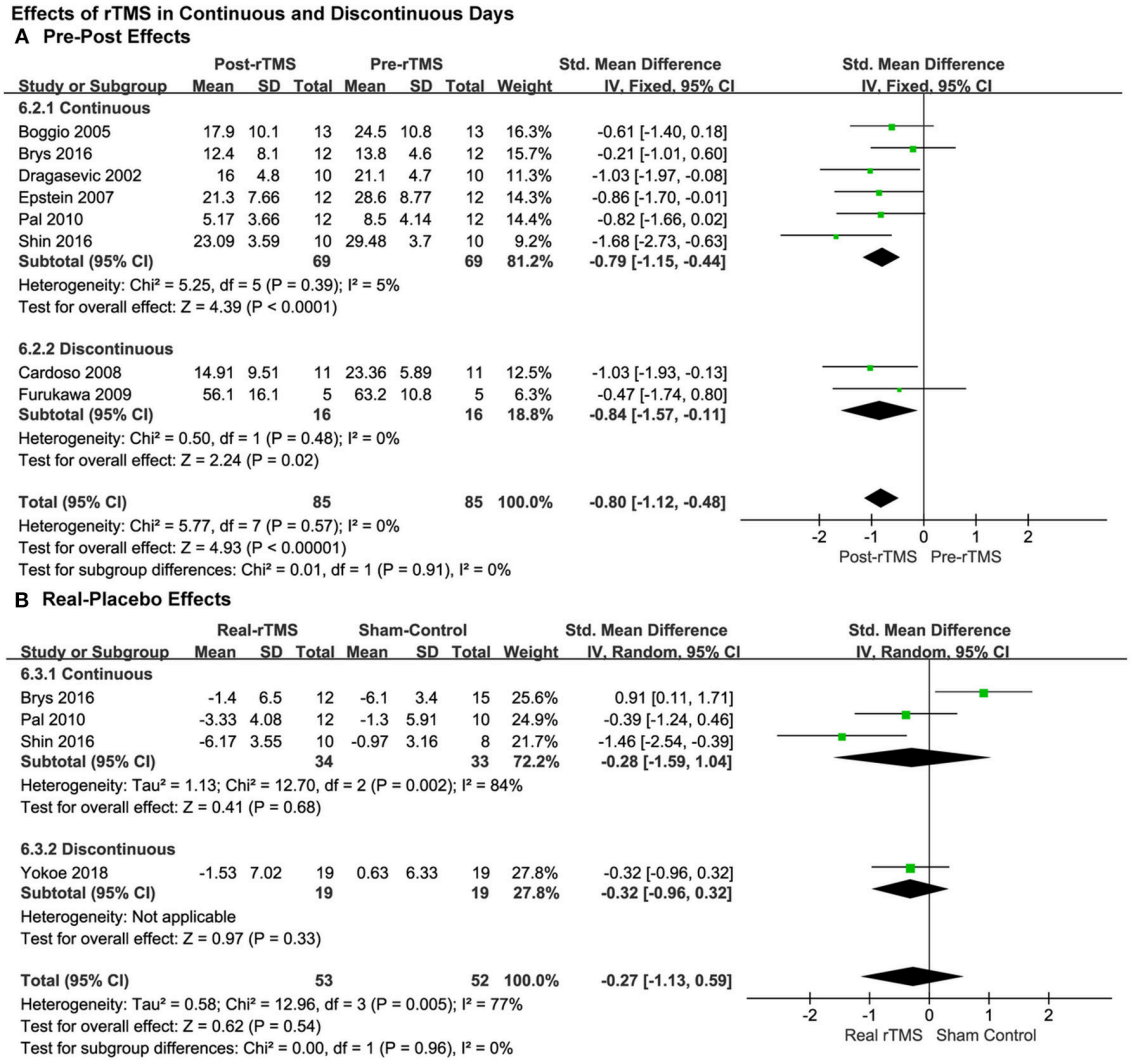
Comparison of the anti-depressive effects of different stimulation models (continuous vs discontinuous) for the studies with pre-post treatment design **(A)** and place-controlled design **(B)**.

Moreover, the results showed significantly therapeutic pre-post effects when the rTMS treatments were carried out both in continuous (SMD = −0.79, 95% CI, −1.15 to −0.44, *P* < 0.0001; Figure 6A) and discontinuous days (SMD = −0.84, 95% CI, −1.57 to −0.11, *P* = 0.02; Figure 6A).

Results from place-controlled studies showed no significant effects of rTMS were applied in continuous or discontinuous days (SMD = −0.28, 95% CI, −1.59 to 1.04, *P* = 0.68, Heterogeneity test: Chi^2^ = 12.7, *P* = 0.002, *I*^2^ = 84%; SMD = −0.32, 95% CI, −0.96 to 0.32, *P* = 0.33; Figure 6B). Therefore, the conclusions should be treated cautiously due to the limited quantity of the studies, lack of adequate RCTs and that placebo effects may also exist.

According to the aforementioned results, we observed that the studies with pre-post design revealed significant effects with no heterogeneity. However, the results of the studies that used placebos as controls, revealed that the effect was not significantly different from placebos. In addition, in the subgroup analyses, a greater effect by rTMS with 90% RMT, 5.0 Hz with discontinuous days was observed compared to the other parameters in both kinds of analyses across the study design.

No significant heterogeneity was observed in the overall and subgroup analysis of pre-post studies, but they existed in all of the analyses of the studies with place-controlled design.

## Discussion

This meta-analysis investigated and supported the anti-depressive effect of rTMS in depressed PD patients when applied to the PFC area. Despite the extensive application of rTMS for MD, few rTMS studies have investigated the effects of different stimulation parameters and assessment methods of rTMS in depressed PD patients so far, and no optimal protocols of rTMS have been established for depressed PD patients. Previously published studies also differ in the efficacy of rTMS therapy due to different parameters used (e.g., intensity and frequency of stimulation, stimulation areas, and pulses per session).

According to the literature (9, 10), hypo-activity in the left DLPFC plays a critical role in the pathophysiology of depression, and normalization of left DLPFC and PFC activity by rTMS has been found to be associated with remission of depression symptoms (11). The DLPFC has been mainly associated with emotional regulation in facial perception (37), and previous studies showed that depressive symptoms could be induced by lesions of left PFC (9, 38). These studies suggest that the moderation of depressive symptoms might be linked to the improvement of activity in the left PFC. Also, two studies have displayed that when compared to normal control groups (19) and PD patients without depression (39), PD patients with depression have a hypo-activity in the left DLPFC. It is expected that similar anti-depressive effects of stimulation of PFC may be produced in depressed PD patients. One reason is that the excitability of the cerebral cortex is speculated to be declined in PD patients due to the change of excitatory inputs from the thalamus, and transcranial magnetic stimulation (TMS) may make up for this declined excitability of the cerebral cortex (40–42). The other reason is that TMS may promote the balance between substantia nigra pars reticulata and internal segment of the globus pallidus. The impact of rTMS on the neurotransmitter is similar to the impact of drug treatment on the neurotransmitter changes from depression in PD patients. This might be one of the effective mechanisms of rTMS treatment of depression in PD patients (15). Boggio reported similar efficacy of HF-rTMS on cognitive function in patients with PD compared to fluoxetine treatment (32). Also, rTMS had a similar efficacy as fluoxetine in increasing cerebral blood flow perfusion in PD patients. It could adjust the excitability of the cortex, improving the circulation of cerebral blood, influencing the metabolism of catecholamine in the brain. It promotes the release of endogenous dopamine, increasing the dopamine around the ipsilateral caudate nucleus, inhibiting the decomposition of dopamine in the brain nervous system and adjusting the excitation of direct loop and indirect loop in the striatum pallidum of the affected side (19).

### Influence of the Frequency on the Effects

Pascual-Leone (43) first used 10.0 Hz rTMS to stimulate the DLPFC of depressed patients in 1995, and his study showed that HF-rTMS and LF-rTMS could differentially alter cortical excitability depending on basal cortical activity (44). Additionally, HF-rTMS is superior to LF-rTMS in relieving the depressive symptoms in PD patients as reported in a meta-analysis (23). Several studies have found that different frequencies of rTMS could induce different changes in local cerebral blood flow (45), the expression of immediate early genes (46) and protein metabolism (47). HF-rTMS may also induce greater changes in brain neurotransmitters such as dopamine (DA), 5-hydroxytryptamine (5-HT), glutamic acid, and brain-derived neurotrophic factor (BDNF) after rTMS treatment (48), as revealed by the increased release of endogenous dopamine in the prefrontal cortex by the SPECT technique (49) as well as increased metabolism of 5-HT/tryptophan in local brain regions after rTMS treatment over left DLPFC (50). High-frequency stimulation activates, whereas low frequency inhibits neural activities which underlie altered regional cortex activity, in turn modulating interactions of different brain regions and the therapeutic reaction to depression (51). Recently a subgroup analysis in a meta-analysis proved that HF-rTMS in frontal regions, containing the supplementary motor area, was not significant, whereas LF-rTMS significantly ameliorated the motor symptoms in PD patients (52). Furthermore, a past study also demonstrated that distinction between the inhibitory and facilitatory effects with different frequencies of rTMS was very hard to achieve (53). Our results found studies that applied less than 1.0, 5.0, and ≥10 Hz of rTMS treatment and produced significant antidepressant pre-post effects to relieve the depressive symptoms in PD. However, the result also showed no significant difference when rTMS was applied at 5 Hz or at greater than or equal to 10.0 Hz when compared to sham stimulation. These differences in therapeutic effects of rTMS on depressed patients with PD need more studies to be verified.

### Influence of the Stimulation Intensity on the Effects

Similar to the prior study reported by Teng (54) that demonstrated a session-dependent therapeutic efficacy of HF-rTMS and with the increase of the HF-rTMS sessions, the efficacy of HF-rTMS in alleviating depressed patients' symptoms also increased. We found that when applied to the supra-threshold of the RMT, rTMS produced significant anti-depressive pre-post treatment effects, which was also observed in the studies with an intensity of 90% RMT, and the effect size was greater than the supra-threshold group. However, the subgroup analysis of studies, which used intensity of 90 or 100% of the RMT, revealed that no significant effect was observed in either of the two groups when compared to sham stimulation. Moreover, some analogous conclusions have been drawn by previous clinical research that the therapeutic effect of rTMS was associated with the different quantity of stimulation sessions on chronic stroke patients (55) and patients with headaches (56). Many factors may be involved, and the mechanisms are still unknown despite a few certain explanations. RMT was defined as the lowest intensity capable of producing motor-evoked potentials (MEPs) of at least 50 μV in 5 out of 10 consecutive trials. In stimulation procedures, the importance of sensation of the skin, which could produce a favorable impact on PD patients was verified by Mally and Stone (57, 58). They revealed that when rTMS was at an intensity of 20% of the RMT, it could improve the symptoms of PD. First, according to Shingo Okabe, the intensity of RMT was not strong enough to induce currents to induce any different structural changes of brain regions under the coil, but it could motivate sensation of the skin, which could produce a potential placebo effect (24). Also, a meta-regression analysis (59) concluded that the efficacy of the long-term UPDRS III scores in PD patients was accompanied by a greater total number of stimulation pulses, and the total number of pulses was unrelated to the effect size of walking performance in PD patients. Hence, we speculate that the increased intensity of the RMT may bring changes in the process of stimulation and generate a better effect for the treatment in various aspects in PD patients. Secondly, disease fluctuation is an inevitable issue when evaluating the effects of a treatment in PD patients. As the illness duration and the time of medication increases, patients will gradually appear to have an on-off phenomenon. This refers to the fluctuation of drug effects after long-term application of levodopa drugs in patients with PD, which is a side effect of such drugs. As for the reason for this phenomenon, it is still not clear, considering that it may be related to the dopamine receptor. The decrease in efficacy and instability are due to changes in dopamine receptors and changes in L-DOPA absorption. Patients may respond differently to the RMT in the different states and these differences in RMT subgroup analysis may possibly be partially due to enrolled patients with heterogeneous illness durations and with variable years of medication in this meta-analysis. Future research is needed to explore this issue.

### Influence of the Day Schedule on the Effects

Our analysis also showed significantly therapeutic pre-post effects from rTMS conducted both in continuous and discontinuous days. Meanwhile, the results also showed no significant effects of rTMS applied in continuous or discontinuous days when compared to sham stimulation. Similar findings have been explored in a double-blind rTMS comparison study made by Szuba et al. (60). They showed that single rTMS sessions can improve mood symptom remission in major depressive patients when rTMS is conducted in twice daily, daily, or three times weekly. Then, Toshiaki Furukawa considered that periodic stimulation over several months may facilitate the reconstruction of the central nervous system (33), cortical excitability may change daily, and intensity of stimulation associated with the RMT may have varied simultaneously (15). Because of this, we speculate that continuous stimulation may induce fatigue of cortical excitability to decease the effect size of stimulation, but according to Epstein CM's hypothesis, patients are mostly debilitated when they are in the “off” condition and the largest efficacy of stimulation could be acquired. Their results partly conform to the presupposition that improvement of motor function along with mood would be found when a focal rTMS treatment is in the left PFC (31). On the other hand, there is a common phenomenon that placebo effects exist in treatment trials of both depression and PD (61), and the rater bias could not be avoided. Therefore, we need more RCTs to illuminate the mechanism of this diversity.

### Limitations

The significantly different effects between frequency, RMT, and treatment in continuous days or discontinuous days should be explained cautiously because of the limited number of studies in this meta-analysis. The main limitation is that these results contain open studies and are not all sham-controlled studies, which may raise several biases. However, no significant difference between the sham-controlled studies compared with non-sham controlled rTMS was found. Additionally, although the current RCTs could minimize the placebo effect, some neuroimaging techniques have demonstrated that sham-rTMS can also produce considerable placebo effects by inducing striatal dopamine release (62, 63). Thus, it is difficult to distinguish if the modest improvement is caused by the placebo effect or not.

In addition, there was a paper published in the American Journal of Epidemiology in 2007 investigating whether meta-analyses of interventions should include observational studies in addition to RCTs. The authors concluded that the advantages of including both observational studies and randomized studies in a meta-analysis would outweigh the disadvantages in many situations and that observational studies should not be excluded (64). In our paper, the number of included randomized trials was small. The non-sham-controlled studies [the study of Epstein et al. (31); Furukawa et al. (33); and Dragasevic et al. (22)] were large intervention efforts and should be useful to inform the design of future randomized trials. Therefore, we have kept these studies. Second, the included studies were relatively lacking in the follow-up period, so we could not evaluate the sustainability of its long-term differentiation. Whether the curative effects of rTMS could be sustained for a long time is still unknown. Third, there may be publication bias due to the fact that the review only applied to English language journals. Fourth, there are substantial diversities in the rTMS protocols, so we were unable to draw definite consequence regarding which mode of rTMS has the optimal parameters to extract from the restrictive quantity of papers. Lastly, our results may have been affected by a few unavoidable factors. Parkinson's patients with depression take medication daily to relieve symptoms and received different DA agonist, SSRIs, and other antidepressant medicines. It would be unethical to leave patients in a state of non-medication treatment and inappropriate to choose a control group (sham rTMS plus placebo drug) without medical treatment. In this way, the participants were kept under constant treatment from DA agonist as well other medications throughout rTMS treatment in most of the studies. This may produce a potential confounding effect and greatly influence rTMS effects. Future work should consider this factor and could explore the effect of rTMS combined with DA agonist/ SSRIs compared with DA agonist/ SSRIs in PD patients.

## Conclusion

rTMS has a significant positive pre-post anti-depressive effect over PFC in patients with depression, especially by using 5.0 Hz frequency with 90% RMT intensity in discontinuous days, which may produce better effects than other parameters. However, the real effect was not different from placebo treatment. Future studies with larger sample size and high-quality studies are required to further corroborate our results and to identify the optimal rTMS protocols.

## Author Contributions

LZ and ZG designed and wrote this study. HP, HC, and MC screened included studies and extracted data. LH, BH, and LX performed the statistical analysis and analyzed the data. GX, FD, and MM contributed to revise the article and modified it. QM reviewed and contributed to the full manuscript.

### Conflict of Interest Statement

GX was employed by company Lotus Biotech.com LLC. The remaining authors declare that the research was conducted in the absence of any commercial or financial relationships that could be construed as a potential conflict of interest.

## References

[1] Poewe W. The natural history of Parkinson's disease. J Neurol. (2006) 253:VII2–6. 10.1007/s00415-006-7002-7 17131223

[2] Lim S-Y Lang AE. The nonmotor symptoms of Parkinson's disease–an overview. Mov Disord. (2010) 25:S123–30. 10.1002/mds.22786 20187234

[3] Reijnders JS Ehrt U Weber WE Aarsland D Leentjens AF. A systematic review of prevalence studies of depression in Parkinson's disease. Mov Disord. (2008) 23:183–9; quiz: 313. 10.1002/mds.21803 17987654

[4] Hely MA Morris JGL Reid WGJ Trafficante R. Sydney multicenter study of Parkinson's disease: non-L-dopa-responsive problems dominate at 15 years. Mov Disord. (2005) 20:190–9. 10.1002/mds.20324 15551331

[5] Brown R Jahanshahi M. Depression in Parkinson's disease: a psychosocial viewpoint. Adv Neurol. (1995) 65:61–84. 7872153

[6] Rektorová I Rektor I Bares M Dostál V Ehler E Fanfrdlová Z. Pramipexole and pergolide in the treatment of depression in Parkinson's disease: a national multicentre prospective randomized study. Eur J Neurol. (2003) 10:399–406. 10.1046/j.1468-1331.2003.00612.x 12823492

[7] Tom T Cummings JL. Depression in Parkinson's disease. Pharmacological characteristics and treatment. Drugs Aging (1998) 12:55–74. 10.2165/00002512-199812010-00006 9467687 PMC5786276

[8] Wagle Shukla A Vaillancourt DE Treatment and physiology in Parkinson's disease and dystonia: using transcranial magnetic stimulation to uncover the mechanisms of action. Curr Neurol Neurosci Rep. (2014) 14:449. 10.1007/s11910-014-0449-5 PMC4171951 24771105

[9] Gainotti G. Emotional behavior and hemispheric side of the lesion. Cortex (1972) 8:41–55. 10.1016/S0010-9452(72)80026-1 5031258

[10] Mottaghy FM Keller CE Gangitano M Ly J Thall M Parker JA. Correlation of cerebral blood flow and treatment effects of repetitive transcranial magnetic stimulation in depressed patients. Psychiatry Res. (2002) 115:1–14. 10.1016/S0925-4927(02)00032-X 12165364

[11] Bench CJ Frackowiak RS Dolan RJ. Changes in regional cerebral blood flow on recovery from depression. Psychol Med. (1995) 25:247–61. 10.1017/S0033291700036151 7675913

[12] Pascual-Leone A Valls-Solé J Wassermann EM Hallett M. Responses to rapid-rate transcranial magnetic stimulation of the human motor cortex. Brain (1994) 117:847–58. 10.1093/brain/117.4.847 7922470

[13] Avery DH Holtzheimer PE III Fawaz W Russo J Neumaier J Dunner DL. A controlled study of repetitive transcranial magnetic stimulation in medication-resistant major depression. Biol. Psychiatry (2006) 59:187–94. 10.1016/j.biopsych.2005.07.003 16139808

[14] Fitzgerald PB Benitez J de Castella A Daskalakis ZJ Brown TL Kulkarni J. A randomized, controlled trial of sequential bilateral repetitive transcranial magnetic stimulation for treatment-resistant depression. Am J Psychiatry (2006) 163:88–94. 10.1176/appi.ajp.163.1.88 16390894

[15] Cardoso EF Fregni F Martins Maia F Boggio PS Luis Myczkowski M Coracini K. rTMS treatment for depression in Parkinson's disease increases BOLD responses in the left prefrontal cortex. Int J Neuropsychopharmacol. (2008) 11:173–83. 10.1017/S1461145707007961 17708780

[16] Rumi DO Gattaz WF Rigonatti SP Rosa MA Fregni F Rosa MO. Transcranial magnetic stimulation accelerates the antidepressant effect of amitriptyline in severe depression: a double-blind placebo-controlled study. Biol Psychiatry (2005) 57:162–6. 10.1016/j.biopsych.2004.10.029 15652875

[17] Chen J Zhou C Wu B Wang Y Li Q Wei Y. Left versus right repetitive transcranial magnetic stimulation in treating major depression: a meta-analysis of randomised controlled trials. Psychiatry Res. (2013) 210:1260–4. 10.1016/j.psychres.2013.09.007 24113125

[18] Mayberg HS Solomon DH. Depression in Parkinson's disease: a biochemical and organic viewpoint. Adv Neurol. (1995) 65:49–60. 7872152

[19] Fregni F Ono CR Santos CM Bermpohl F Buchpiguel C Barbosa ER. Effects of antidepressant treatment with rTMS and fluoxetine on brain perfusion in PD. Neurology (2006) 66:1629–37. 10.1212/01.wnl.0000218194.12054.60 16769932

[20] Fregni F Santos CM Myczkowski ML Rigolino R Gallucci-Neto J Barbosa ER. Repetitive transcranial magnetic stimulation is as effective as fluoxetine in the treatment of depression in patients with Parkinson's disease. J Neurol Neurosurg Psychiatr. (2004) 75:1171–4. 10.1136/jnnp.2003.027060 15258224 PMC1739189

[21] Shin HW Youn YC Chung SJ Sohn YH. Effect of high-frequency repetitive transcranial magnetic stimulation on major depressive disorder in patients with Parkinson's disease. J Neurol. (2016) 263:1442–8. 10.1007/s00415-016-8160-x 27178002

[22] Dragasevic N Potrebic A Damjanovic A Stefanova E Kostic VS. Therapeutic efficacy of bilateral prefrontal slow repetitive transcranial magnetic stimulation in depressed patients with Parkinson's disease: an open study. Mov Disord. (2002) 17:528–32. 10.1002/mds.10109 12112202

[23] Xie CL Chen J Wang XD Pan JL Zhou Y Lin SY. Repetitive transcranial magnetic stimulation (rTMS) for the treatment of depression in Parkinson disease: a meta-analysis of randomized controlled clinical trials. Neurol Sci. (2015) 36:1751–61. 10.1007/s10072-015-2345-4 26209930

[24] Okabe S Ugawa Y Kanazawa I. 0.2-Hz repetitive transcranial magnetic stimulation has no add-on effects as compared to a realistic sham stimulation in Parkinson's disease. Mov Disord. (2003) 18:382–8. 10.1002/mds.10370 12671943

[25] Wang HJ Tan G Zhu LN Chen D Xu D Chu SS The efficacy of repetitive transcranial magnetic stimulation for Parkinson disease patients with depression. Int J Neurosci. (2018) 9:1–24. 10.1080/00207454.2018.1495632 29985089

[26] Moher D Schulz KF Altman D. The CONSORT statement: revised recommendations for improving the quality of reports of parallel-group randomized trials. JAMA (2001) 285:1987–91. 10.1001/jama.285.15.1987 11308435

[27] Higgins JPT Green S Cochrane Handjournal for Systematic Reviews of Interventions Version 5.1.0: The Cochrane Collaboration (2011). Available online at: http:www.cochrane-handjournal.org

[28] Portney LG Watkins MP Foundations of Clinical Research, IIIrd Edition. Toronto, ON: Pearson International Edition (2009).

[29] Zintzaras E Ioannidis JP. HEGESMA: genome search meta-analysis and heterogeneity testing. Bioinformatics (2005) 21:3672–3. 10.1093/bioinformatics/bti536 15955784

[30] Sedgwick P Meta-analyses: how to read a funnel plot. BMJ (2013) 346:f1342 10.1136/bmj.f1342 26377337

[31] Epstein CM Evatt ML Funk A Girard-Siqueira L Lupei N Slaughter L. An open study of repetitive transcranial magnetic stimulation in treatment-resistant depression with Parkinson's disease. Clin Neurophysiol. (2007) 118:2189–94. 10.1016/j.clinph.2007.07.010 17714987 PMC2121115

[32] Boggio PS Fregni F Bermpohl F Mansur CG Rosa M Rumi DO. Effect of repetitive TMS and fluoxetine on cognitive function in patients with Parkinson's disease and concurrent depression. Mov Disord. (2005) 20:1178–84. 10.1002/mds.20508 15895421

[33] Furukawa T Izumi S-I Toyokura M Masakado Y. Effects of low-frequency repetitive transcranial magnetic stimulation in Parkinson's disease. Tokai J Exp Clin Med. (2009) 34:63–71. 21319001

[34] Pal E Nagy F Aschermann Z Balazs E Kovacs N. The impact of left prefrontal repetitive transcranial magnetic stimulation on depression in Parkinson's disease: a randomized, double-blind, placebo-controlled study. Mov Disord. (2010) 25:2311–7. 10.1002/mds.23270 20740485

[35] Brys M Fox MD Agarwal S Biagioni M Dacpano G Kumar P. Multifocal repetitive TMS for motor and mood symptoms of Parkinson disease: a randomized trial. Neurology (2016) 87:1907–15. 10.1212/WNL.0000000000003279 27708129 PMC5100715

[36] Yokoe M Mano T Maruo T Hosomi K Shimokawa T Kishima H. The optimal stimulation site for high-frequency repetitive transcranial magnetic stimulation in Parkinson's disease: a double-blind crossover pilot study. J Clin Neurosci. (2018) 47:72–8. 10.1016/j.jocn.2017.09.023 29054329

[37] Leppänen J. Emotional information processing in mood disorders: a review of behavioral and neuroimaging findings. Curr Opin Psychiatry (2006) 19:34–9. 10.1097/01.yco.0000191500.46411.00 16612176

[38] Gasparrini W Satz P Heilman K Coolidge F. Hemispheric asymmetries of affective processing as determined by the Minnesota multiphasic personality inventory. J Neurol Neurosurg Psychiatr. (1978) 41:470–3. 10.1136/jnnp.41.5.470 660213 PMC493058

[39] Ring H Bench C Trimble M Brooks D Frackowiak R Dolan R. Depression in Parkinson's disease. A positron emission study. Br J Psychiatry (1994) 165:333–9. 10.1192/bjp.165.3.333 7994502

[40] Alexander G DeLong M Strick P. Parallel organization of functionally segregated circuits linking basal ganglia and cortex. Annu Rev Neurosci. (1986) 9:357–81. 10.1146/annurev.ne.09.030186.002041 3085570

[41] Alexander G Crutcher M. Functional architecture of basal ganglia circuits: neural substrates of parallel processing. Trends Neurosci. (1990) 13:266–71. 10.1016/0166-2236(90)90107-L 1695401

[42] DeLong M. Primate models of movement disorders of basal ganglia origin. Trends Neurosci. (1990) 13:281–5. 10.1016/0166-2236(90)90110-V 1695404

[43] Pascual-Leone A Rubio B Pallardó F Catalá MD. Rapid-rate transcranial magnetic stimulation of left dorsolateral prefrontal cortex in drug-resistant depression. Lancet (1996) 348:233–7. 10.1016/S0140-6736(96)01219-6 8684201

[44] Siebner HR Lang N Rizzo V Nitsche MA Paulus W Lemon RN. Preconditioning of low-frequency repetitive transcranial magnetic stimulation with transcranial direct current stimulation: evidence for homeostatic plasticity in the human motor cortex. J Neurosci. (2004) 24:3379–85. 10.1523/JNEUROSCI.5316-03.2004 15056717 PMC6730024

[45] Speer AM Benson BE Kimbrell TK Wassermann EM Willis MW Herscovitch P. Opposite effects of high and low frequency rTMS on mood in depressed patients: relationship to baseline cerebral activity on PET. J Affect Disord. (2009) 115:386–94. 10.1016/j.jad.2008.10.006 19027962 PMC2779113

[46] Aydin-Abidin S Trippe J Funke K Eysel UT Benali A. High- and low-frequency repetitive transcranial magnetic stimulation differentially activates c-Fos and zif268 protein expression in the rat brain. Exp Brain Res. (2008) 188:249–61. 10.1007/s00221-008-1356-2 18385988

[47] Houdayer E Degardin A Cassim F Bocquillon P Derambure P Devanne H. The effects of low- and high-frequency repetitive TMS on the input/output properties of the human corticospinal pathway. Exp Brain Res. (2008) 187:207–17. 10.1007/s00221-008-1294-z 18259738

[48] Brunelin J Poulet E Boeuve C Zeroug-vial H d'Amato T Saoud M. Efficacy of repetitive transcranial magnetic stimulation (rTMS) in major depression: a review. Encephale (2007) 33:126–34. 10.1016/S0013-7006(07)91542-0 17675907

[49] Pogarell O Koch W Popperl G Tatsch K Jakob F Zwanzger P Striatal dopamine release after prefrontal repetitive transcranial magnetic stimulation in major depression: preliminary results of a dynamic [123I] IBZM SPECT study. J Psychiatr Res. (2006) 40:307–14. 10.1016/j.jpsychires.2005.09.001 16259998

[50] Sibon I Strafella AP Gravel P Ko JH Booij L Soucy JP. Acute prefrontal cortex TMS in healthy volunteers: effects on brain 11C-alphaMtrp trapping. Neuroimage (2007) 34:1658–64. 10.1016/j.neuroimage.2006.08.059 17188517

[51] Peng H Zheng H Li L Liu J Zhang Y Shan B. High-frequency rTMS treatment increases white matter FA in the left middle frontal gyrus in young patients with treatment-resistant depression. J Affect Disord. (2012) 136:249–57. 10.1016/j.jad.2011.12.006 22217432

[52] Chou Y Hickey P Sundman M Song A Chen N. Effects of repetitive transcranial magnetic stimulation on motor symptoms in Parkinson disease: a systematic review and meta-analysis. JAMA Neurol. (2015) 72:432–40. 10.1001/jamaneurol.2014.4380 25686212 PMC4425190

[53] Demirtas-Tatlidede A Vahabzadeh-Hagh A Pascual-Leone A. Can noninvasive brain stimulation enhance cognition in neuropsychiatric disorders? Neuropharmacology (2013) 64:566–78. 10.1016/j.neuropharm.2012.06.020 22749945 PMC3725288

[54] Teng S Guo Z Peng H Xing G Chen H He B. High-frequency repetitive transcranial magnetic stimulation over the left DLPFC for major depression: session-dependent efficacy: a meta-analysis. Eur Psychiatry (2017) 41:75–84. 10.1016/j.eurpsy.2016.11.002 28049085

[55] Kim Y Jung J Shin S. A comparison of the effects of repetitive transcranial magnetic stimulation (rTMS) by number of stimulation sessions on hemispatial neglect in chronic stroke patients. Exp Brain Res. (2015) 233:283–9. 10.1007/s00221-014-4112-9 25332169

[56] Kalita J Laskar S Bhoi S Misra U. Efficacy of single versus three sessions of high rate repetitive transcranial magnetic stimulation in chronic migraine and tension-type headache. J Neurol. (2016) 263:2238–46. 10.1007/s00415-016-8257-2 27541044

[57] Mally J Stone TW. Improvement in Parkinsonian symptoms after repetitive transcranial magnetic stimulation. J Neurol Sci. (1999) 162:179–84. 10.1016/S0022-510X(98)00318-9 10202984

[58] Mally J Stone TW. Therapeutic and “dose-dependent” effect of repetitive microelectroshock induced by transcranial magnetic stimulation in Parkinson's disease. J Neurosci Res. (1999) 57:935–40. 10467265

[59] Chung CL Mak MKY. Effect of repetitive transcranial magnetic stimulation on physical function and motor signs in parkinson's disease: a systematic review and meta-analysis. Brain Stimul. (2016) 9:475–87. 10.1016/j.brs.2016.03.017 27117282

[60] Szuba M O'Reardon J Rai A Snyder-Kastenberg J Amsterdam J Gettes D. Acute mood and thyroid stimulating hormone effects of transcranial magnetic stimulation in major depression. Biol Psychiatry (2001) 50:22–7. 10.1016/S0006-3223(00)01118-5 11457420

[61] Goetz CG Leurgans S Raman R Stebbins GT. Objective changes in motor function during placebo treatment in PD. Neurology (2000) 54:710–4. 10.1212/WNL.54.3.710 10680808

[62] Strafella AP Ko JH Monchi O. Therapeutic application of transcranial magnetic stimulation in Parkinson's disease: the contribution of expectation. Neuroimage (2006) 31:1666–72. 10.1016/j.neuroimage.2006.02.005 16545582 PMC2967525

[63] Kim JY Chung EJ Lee WY Shin HY Lee GH Choe YS. Therapeutic effect of repetitive transcranial magnetic stimulation in Parkinson's disease: analysis of [11C] raclopride PET study. Mov Disord. (2008) 23:207–11. 10.1002/mds.21787 17999422

[64] Shrier I Boivin J Steele R Platt R Furlan A Kakuma R. Should meta-analyses of interventions include observational studies in addition to randomized controlled trials? A critical examination of underlying principles. Am J Epidemiol. (2007) 166:1203–9. 10.1093/aje/kwm189 17712019

